# Mental health, suicidal behavior and sexual orientation in Portugal

**DOI:** 10.1192/j.eurpsy.2021.1555

**Published:** 2021-08-13

**Authors:** H. Pereira

**Affiliations:** 1 Ubi, Research Centre in Sports Sciences, Health Sciences and Human Development, Covilha, Portugal; 2 Psychology And Education, University of Beira Interior, Covilha, Portugal; 3 Cics-ubi, Centre for Research in Health Sciences, Covilha, Portugal

**Keywords:** Suicide Behavior, Depression, Anxiety, Sexual orientation

## Abstract

**Introduction:**

Sexual minority individuals consistently report higher rates of mental disorders and suicidal behavior than heterosexuals. However, much of this research is limited to Anglo-Saxon studies and no information on Portuguese reality is available.

**Objectives:**

The purpose of this study is to compare levels of mental functioning and suicidal behavior among heterosexual, bisexual, and homosexual individuals in Portugal.

**Methods:**

Using online surveys, 1140 individuals (62.40% women, Mage = 36.83, SDage = 13.39, 76.4% heterosexual, 9.4% bisexual, and 14.2% gay or lesbian) completed the BSI subscales for depression and anxiety symptoms, as well as the Suicide Behaviors Questionnaire-Revised.

**Results:**

Self-identified bisexual participants presented higher levels of depressive and anxiety symptoms and higher levels of suicidal ideation and likelihood of suicidal behavior than homosexual and heterosexual participants (who scored the lowest); yet, homosexual participants showed higher levels of suicide attempts. Also, depression and anxiety symptoms were strongly and positively correlated with all dimensions of suicidal behavior. Finally, hierarchical multiple regression analysis showed that higher levels of depression and non-heterosexual sexual orientations were significant predictors of suicidal ideation and the likelihood of suicidal behavior.

**Conclusions:**

The present study adds to the evidence that sexual minority individuals are at risk of increased mental health problems and suicidal behavior, compared to heterosexuals, and reiterates the need for local political and legislative efforts to normalize LGB identities, fighting continued institutional heterosexism, interpersonal intolerance. Mental health providers and mental health policymakers need to consider these results if they want to address inequalities in mental health and in suicidality among these minority groups.

